# Modeling the differences in the time-series profiles of new COVID-19 daily confirmed cases in 3,108 contiguous U.S. counties: A retrospective analysis

**DOI:** 10.1371/journal.pone.0242896

**Published:** 2021-11-03

**Authors:** Fadel M. Megahed, L. Allison Jones-Farmer, Longwen Zhao, Steven E. Rigdon

**Affiliations:** 1 Farmer School of Business, Miami University, Oxford, OH, United States of America; 2 College for Public Health and Social Justice, Saint Louis University, Saint Louis, MO, United States of America; The University of Tennessee Knoxville, UNITED STATES

## Abstract

**Objective:**

The COVID-19 pandemic in the U.S. has exhibited a distinct multiwave pattern beginning in March 2020. Paradoxically, most counties do not exhibit this same multiwave pattern. We aim to answer three research questions: (1) How many distinct clusters of counties exhibit similar COVID-19 patterns in the time-series of daily confirmed cases? (2) What is the geographic distribution of the counties within each cluster? and (3) Are county-level demographic, socioeconomic and political variables associated with the COVID-19 case patterns?

**Materials and methods:**

We analyzed data from counties in the U.S. from March 1, 2020 to January 2, 2021. Time series clustering identified clusters in the daily confirmed cases of COVID-19. An explanatory model was used to identify demographic, socioeconomic and political variables associated with the outbreak patterns.

**Results:**

Three patterns were identified from the cluster solution including counties in which cases are still increasing, those that peaked in the late fall, and those with low case counts to date. Several county-level demographic, socioeconomic, and political variables showed significant associations with the identified clusters.

**Discussion:**

The pattern of the outbreak is related both to the geographic location within the U.S. and several variables including population density and government response.

**Conclusion:**

The reported pattern of cases in the U.S. is observed through aggregation of the daily confirmed COVID-19 cases, suggesting that local trends may be more informative. The pattern of the outbreak varies by county, and is associated with important demographic, socioeconomic, political and geographic factors.

## Background and significance

**Fig 1:**
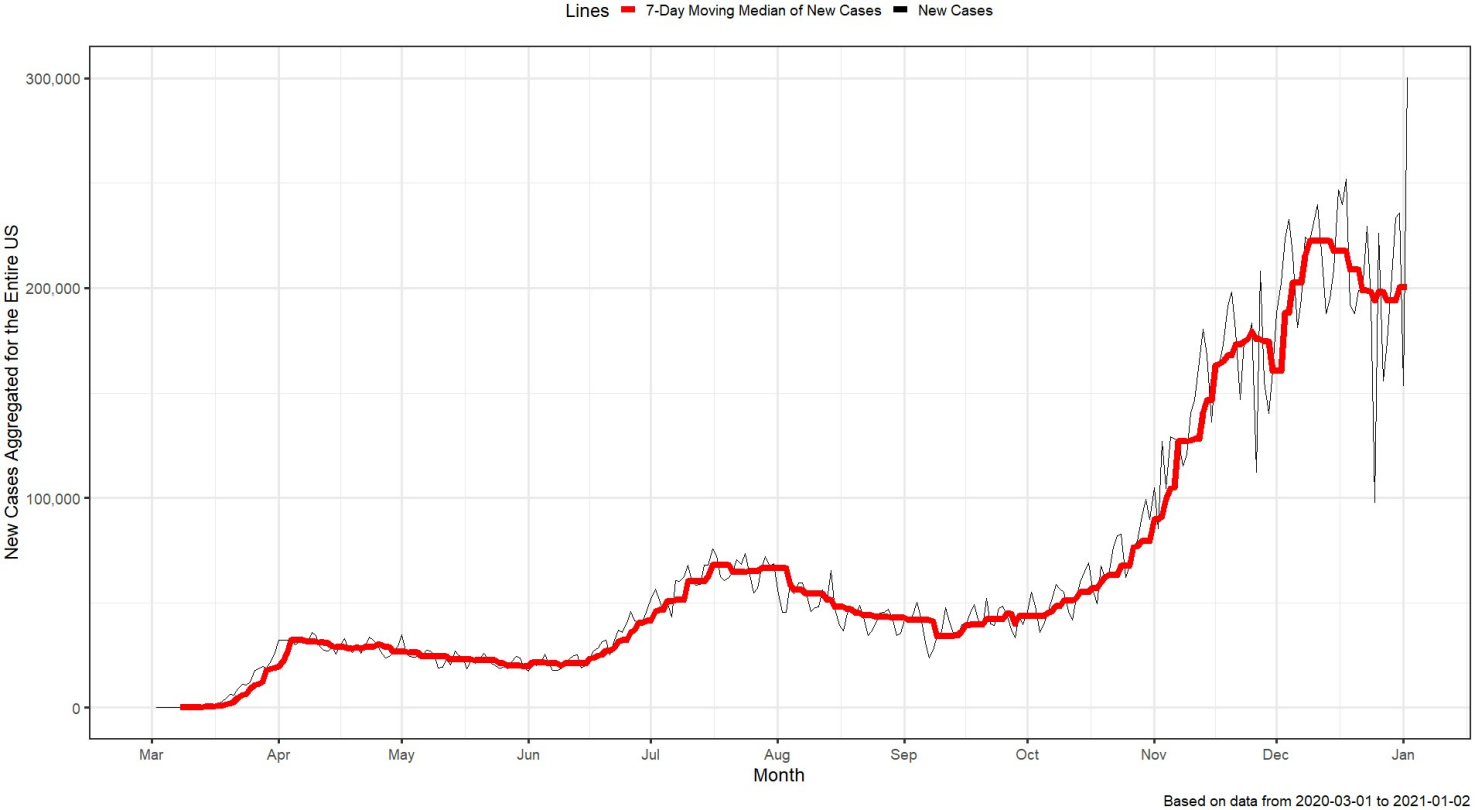
New COVID-19 cases for the entire US. The black and red lines correspond to the reported daily new cases, and the 7-day moving median of daily new cases, respectively.

The daily number of U.S. COVID-19 cases, which we present in Fig 1, shows that there have been three distinct waves. This observation has also been made in the *National Strategy for the COVID-19 Response and Pandemic Preparedness* prepared by the Biden administration [1, p. 24]. The first wave began in March 2020, peaked in April, and then receded somewhat following a widespread lockdown. The number of cases began to rise again in early June once states began to reopen. With further restrictions and health guidelines, the cases seemed to recede by the end of July; however, there was a substantial increase in new cases between late fall 2020 and the end of the study period. In terms of number of reported COVID-19 cases, the second wave was larger than the first and the third wave has been much larger than the first two. Paradoxically, most counties in the U.S. have not exhibited the same multi-wave pattern seen in the aggregated U.S. data. Many counties, especially in the Northeast, exhibited a large first wave followed by a smaller second wave. On the other hand, many counties in the Midwest exhibited a small first wave followed by a larger second wave in terms of cases. As of late fall 2020, most counties in the U.S. saw a resurgence in the number of cases; however, some counties, especially small rural ones, did not exhibit any increases in the number of reported COVID-19 cases until late fall (as of the end of 2020).

**Fig 2:**
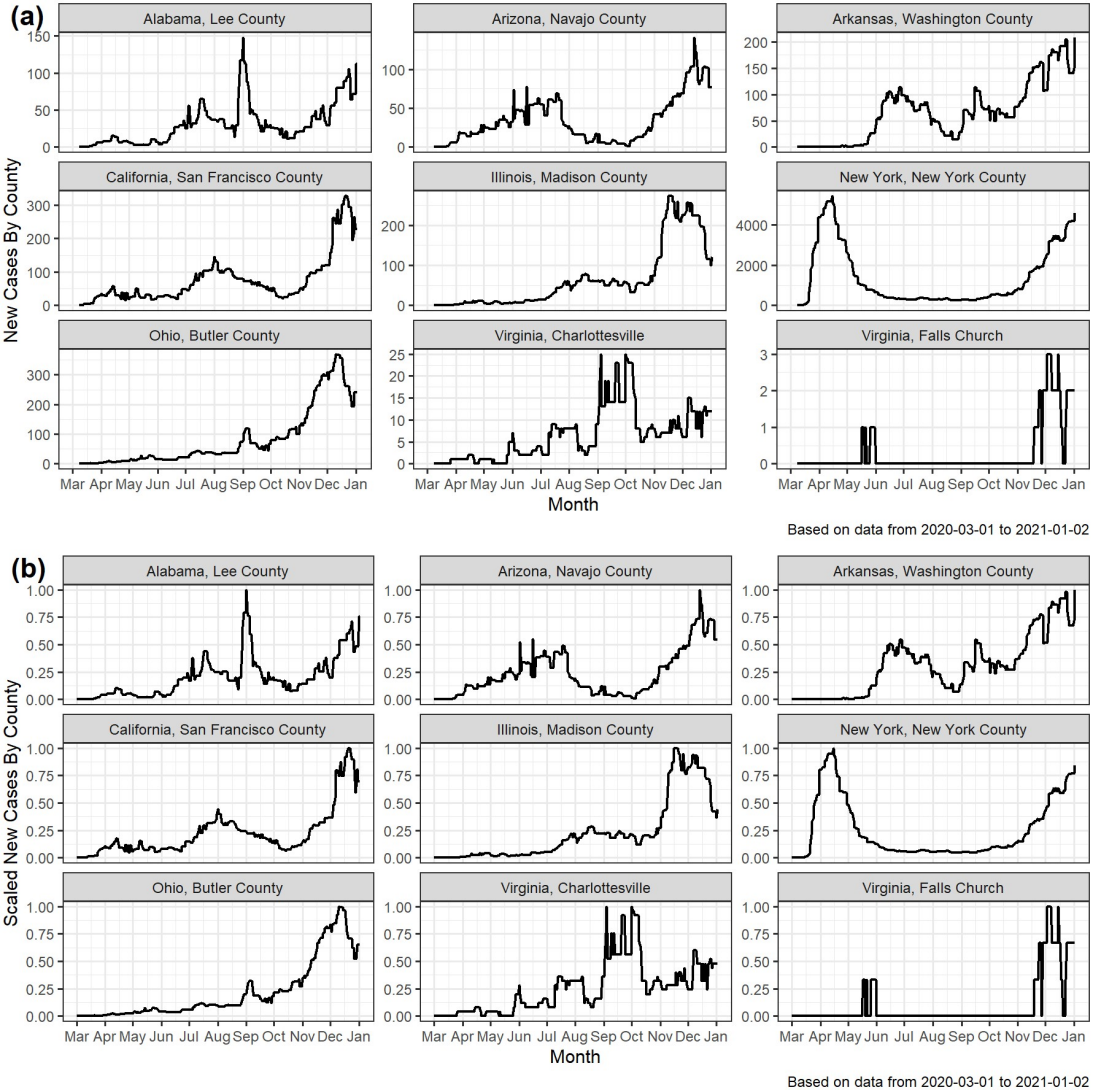
The 7-day moving medians of the (a) number of new daily cases, and (b) scaled [0-1] daily cases for a sample of nine U.S. counties.

Fig 2a. shows the 7-day moving medians for the number of new cases in a sample of nine U.S. counties. The number of cases varied greatly among these counties. To visualize the shape (as opposed to the magnitudes) of the outbreaks, we scaled each time series so that the maximum value is 1. The scaled time series are shown in Fig 2b. In New York County, NY, we see a large early wave, and a second large wave that began in October. In Madison County, IL (a Midwestern county near St. Louis, MO), a sustained increase in cases is observed in the late summer, followed by a surge in cases in the late fall. In Butler County, OH there has been an increasing trend in confirmed cases since late summer. Note that throughout this paper, we use the term county to represent counties, parishes, and independent cities (e.g., several cities within Virginia are incorporated as independent and Louisiana uses the term parish instead of county).

Although many studies of the COVID-19 pandemic are emerging, there has been some systematic evidence worldwide that, in addition to geography, several important demographic, socioeconomic, and political variables may be associated with COVID-19 case patterns and outcomes. Many of these studies have been conducted at the country-level or in countries outside of the U.S. For example, Ficetola and Rublini [2] studied the relationship between environmental effects, containment measures, and early COVID-19 outbreaks at the country level. They showed that the government containment measures and some country-level demographics such as the per capita government health expenditures swamped the effect of environmental variables such as temperature and humidity that are typically associated with disease outbreaks. While there have been some media reports of reduced COVID-19 cases in rural locations [3], other reports suggest that the COVID-19 outcomes such as deaths may be higher in rural locations [4, 5]. In addition, several reports suggest that the population size [6], the Population Density [2] as well as the movement of the population [6] affects the COVID-19 outbreak.

According to the Centers for Disease Control and Prevention (CDC), eight out of ten COVID-19 related deaths in the U.S. have been in adults age 65 years or older, and those in the 65-74 year old group are 1,100 times more likely to die of the disease compared to 5-17 year olds [7]. Although it is clear that once contracted, older adults typically experience more severe outcomes from the COVID-19 virus, it is unclear how the age of the population in a region relates to the spread of the virus as measured by the number of cases. Wu et al. [8] showed that susceptibility to COVID-19 increased with age, and those over 60 were twice as susceptible as those aged 30-59. In contrast, Brown et al. [9] studied the COVID-19 outbreak at the U.S. county-level and suggested that counties with a higher proportion of the population over aged 65 have fewer cases. This counter-intuitive relationship may be due, in part, to the complex interplay of the regional demographics that include the population density, the poverty level, and other variables. For example, Jung et al. [10] showed that there was a U-shaped relationship between the poverty percentile in a U.S. county and the number of COVID-19 cases. Counties with both lower and higher poverty percentiles in the poverty distribution among counties experienced higher case counts early in the pandemic, while those near the median experienced lower case counts. Brown et al. [9] also considered the relationship between the poverty level and COVID-19 case counts at the U.S. county level. They showed that the poverty level did not have much effect once they controlled for state-level effects in a hierarchical model.

Other research suggests that the relationship between the COVID-19 outbreak and the socioeconomic makeup of a region may be more nuanced. Papageorge et al. [11] found that income was strongly associated with compliance and protective behavioral responses to the outbreak, with poorer individuals less able to practice these behaviors such as social distancing due, in part, to the nature of their occupations. Similar results were found by Ferdous et al. [12] and Zhong et al. [13] who showed that adherence to prevention practices such as social distancing and mask wearing was positively associated with age, income, and occupational status.

Several studies suggest a strong relationship between government containment measures and reductions in COVID-19 cases at the country-level. The Blavatnick School of Government [14] has developed an index to measure the containment and closures response at the national and subnational level for several countries. Ficetola and Rubolini [2] showed that increased government containment as measured by this index was associated with reduced COVID-19 outbreak. Similarly, Islam et al. [15] also showed that implementation of any of a list of interventions such as school closures or limitations on mass gatherings was associated with reduced incidence of COVID-19 at the country level.

The observed local-level patterns along with the regional demographics and governmental response information can be used to inform decisions made to mitigate the pandemic. Recently, Bakken [16] argued that “informatics is a critical strategy in combating the COVID-19 pandemic.” She lists five health informatics practice domains, one of which is “enhancing health decision making, processes, and outcomes.” The roles of the federal and local governments in enacting measures like school closures and business restrictions to combat the virus have been under debate. Not everyone agrees on how decisions should be made. For example, Koh [17] argues that there should be one strategy, not 50. Similarly, Haffajee and Mello [18] argue that “strong, decisive national action is therefore imperative.” On the other side of the debate, others, such as Davidson [19] argue that the federal government does not have the authority to enact measures like lockdowns, because these powers are reserved for the states. The arguments for or against federal vs. state vs. local control of mitigation standards may be clarified by a better understanding of the pattern of outbreaks.

## Objective

The observation that most counties do not follow the pattern of the aggregated number of reported COVID-19 cases in the U.S. along with the emerging research that suggests relationships between regional demographic, socioeconomic and political variables to the emergence of COVID-19 cases led us to pose these research questions:

1. How many distinct clusters of counties exhibit similar COVID-19 patterns in the time-series of daily confirmed cases?
2. What is the geographic distribution of the counties within each cluster?
3. Are county-level demographic, socioeconomic and political variables associated with the COVID-19 case patterns?

## Materials and methods

To explore these research questions we used a time series cluster analysis of counties within the contiguous U.S. to identify groups of counties with similar COVID-19 outbreak patterns. A visualizaton of the cluster solution provides information on the distribution of the cluster patterns across the U.S. Finally, we used a multinomial regression model to identify county-level variables that are associated with the observable variation in the COVID-19 outbreak patterns.

This analysis was conducted in three stages. In Stage 0, county-level data were gathered from several sources, merged, and preprocessed for consistency. In Stage 1, time series clustering was performed on the number of newly reported confirmed COVID 19 cases per day. Finally, in Stage 2, a multinomial regression model was fit to describe the relationship between cluster membership and several demographic, socioeconomic and political factors describing the counties.

### Stage 0: Data acquisition and preprocessing

Guidoti and Ardia [20], provide an open-source COVID-19 data hub to facilitate research regarding the novel coronavirus. Data were completely anonymous. The disease outbreak was declared a pandemic by the World Health Organization (WHO) on March 11, 2020 and a national emergency by the U.S. on March 13, 2020. To capture the progression of disease in the U.S., the number of confirmed COVID-19 cases at the county-level from March 1, 2020 through January 2, 2021 was extracted from the COVID-19 data hub [20]. The original source files for confirmed COVID-19 cases in the data hub is the *Johns Hopkins Center for Systems Science and Engineering* [21]. The chosen dates were selected to capture full epidemiological weeks, resulting in 44 epidemiological weeks capturing how the outbreak has evolved in different U.S. counties in (mostly) 2020. Only data from the contiguous 48 states were included in our analysis. The number of newly reported confirmed COVID-19 cases per day is used to establish the pattern of the pandemic’s progression in each county, and is the only information used to determine the time series clustering of the counties.

**Fig 3:**
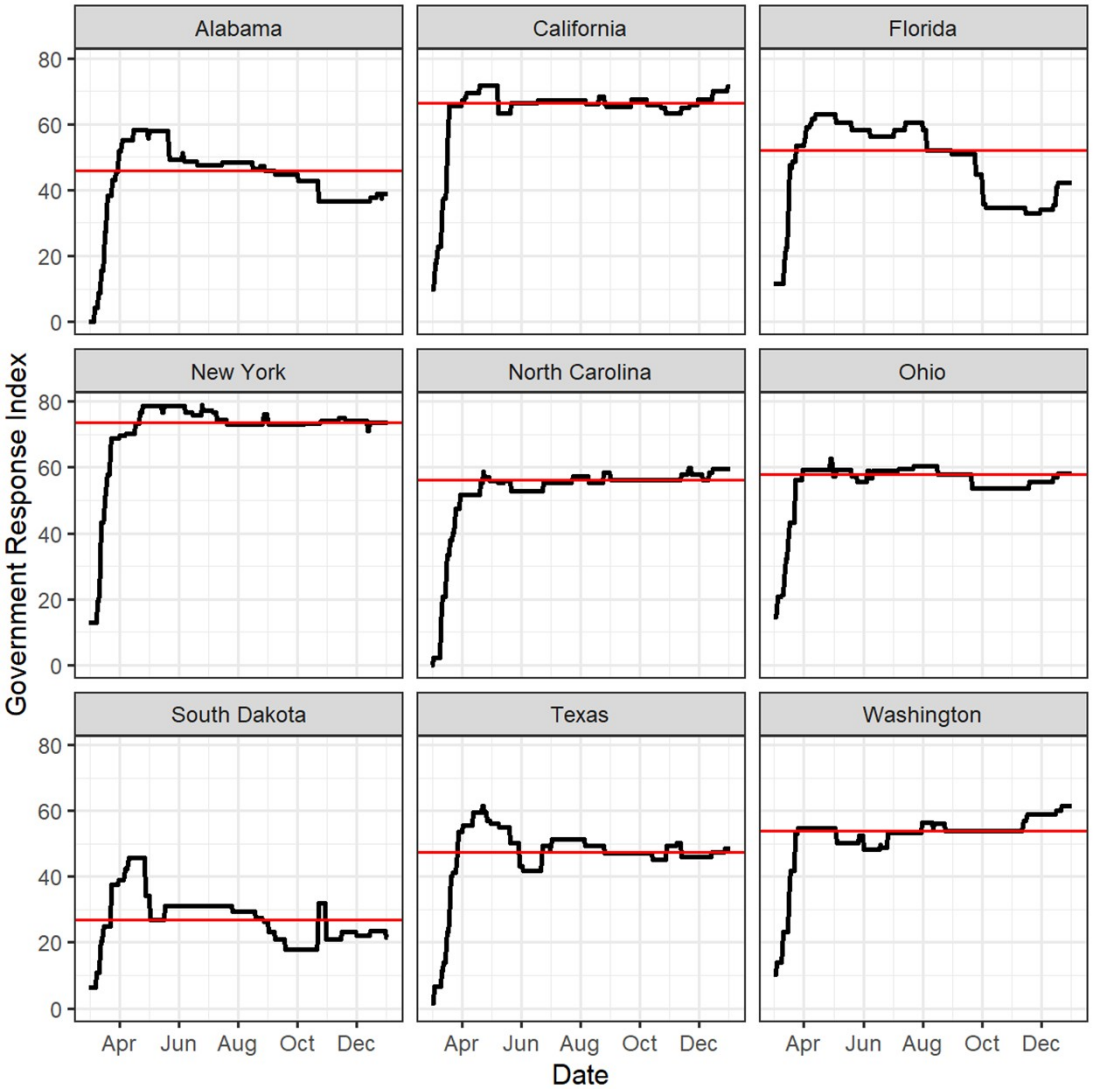
The Blavatnik School of Government’s Government Response Index for nine representative states. The median index is shown in red.

**Fig 4:**
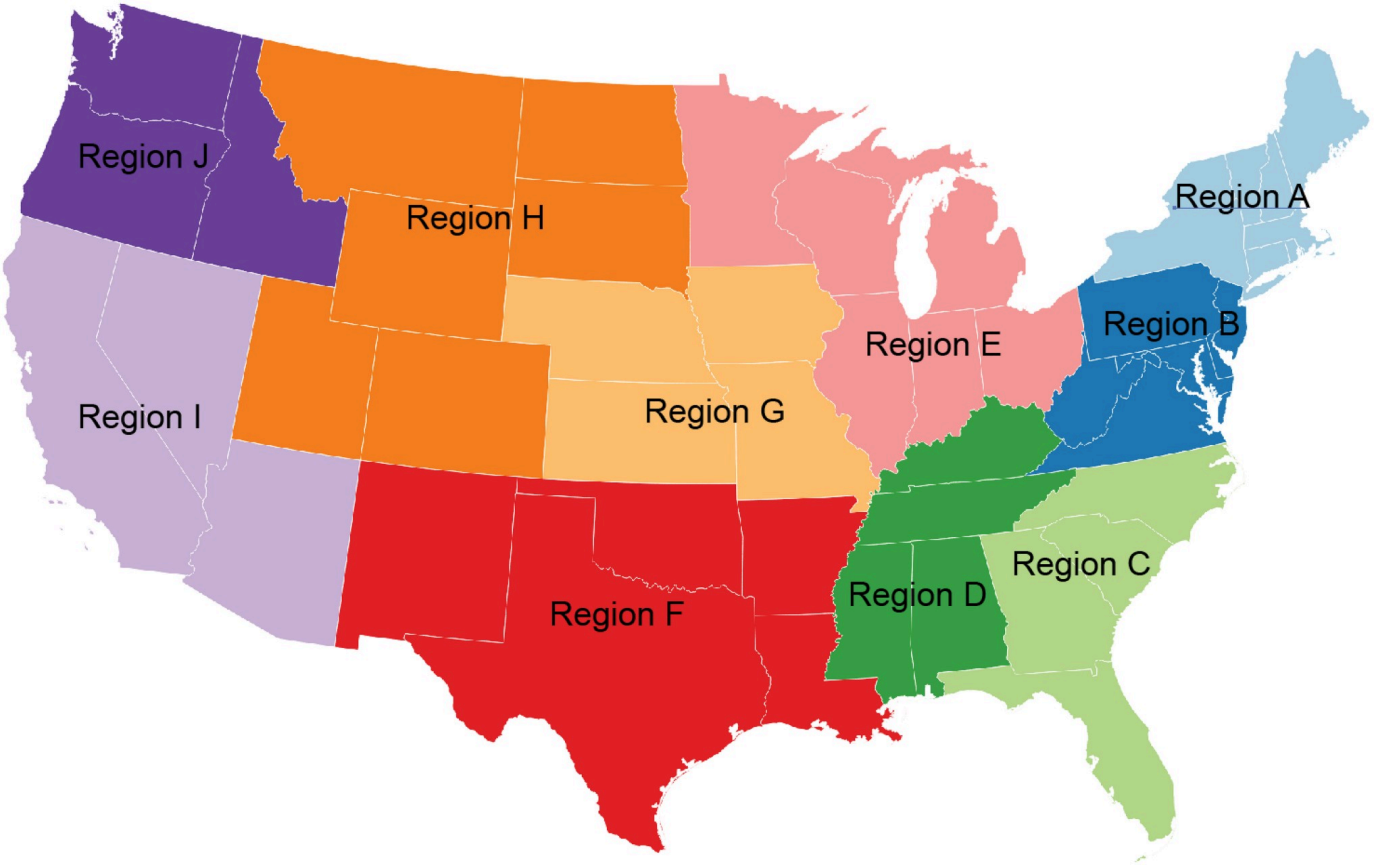
The CDC’s 10 region framework for chronic disease prevention and health promotion. Documentation for these regions can be found at [28].

Additional county-level exogenous variables were extracted from several sources to be used in an explanatory model to describe the clusters of outbreak patterns. The variables include the following:

- ***Rural/Underserved Counties***: The Consumer Financial Bureau [22] provides an annual list of rural or underserved counties to help creditors determine which properties are located in rural or underserved areas. This list was selected to capture the the relationship of the macroeconomic and socio-economic factors to the patterns of the COVID-19 outbreak at the county level in the U.S. A county is designated as rural if the United States Department of Agriculture Economic Research Service assigns it an Urban Influence Code of 4, 6, 7, 8, 9, 10, 11, or 12. More information about Urban Influence Codes can be found at [23]. A county is underserved if, according to the Home Mortgage Disclosure Act [22] it has no more than two creditors extended coverage transactions secured by first liens in the county five times or more.
- ***Population Density***: Based on the US Census Data [24], the land area in square miles for each county and the population of each county, both from 2011, were extracted. The population and the land area were combined to compute the county’s *Population Density*. Due to skewness in this variable, the natural logarithm of *Population Density* is used in the explanatory model.
- ***Percent Seniors***: From Kaiser Health News [25], county-level information was extracted on the percent of population aged 60+. This data set was originally gathered by Kaiser Health News for an analysis of hospital cost reports filed to the Centers for Medicare & Medicaid Services.
- ***Poverty***: Based on the U.S. Census’s Small Area Income and Poverty Estimates (SAIPE) Program [26], the percent of population in poverty for each county was extracted. The estimate is based on 2018 data (released in December 2019). At the time of the start of our analysis, these estimates were the most up to date publicly available data.
- ***Government Response***: The overall *Government Response Index* (at the U.S. state level) from the Blavatnik School of Government [14] was downloaded on February 5, 2021. The methodology for computing the index is described at the Oxford University Covid-19 Tracker Github [27]. This index includes the following:
  - Closures such as school or workplace closures
  - Economic Response such as income support and debt relief
  - Health Systems such as testing, contact tracing, and vaccine availability
  Higher values of the Government Response Index indicate stronger government response and containment measures related to the pandemic. The Government Response Index changes over time during the study period. As shown in Fig 3, the index ramped up in early spring 2020 and for most states the index leveled off at a near constant value during the course of the study period. Thus, we summarized the index using the median value over the study period, which approximates the Government Response Index over the majority of the study period.
- ***Region***: Using the CDC’s 10 Region Framework for Chronic Disease Prevention and Health Promotion [28], geographic region indicators were obtained. Fig 4 shows these ten regions. This source of defining regions within the U.S. was selected because the regions were developed by the CDC’s National Center for Chronic Disease Prevention and Health Promotion (NCCDPHP) to promote consistency in communications and technical assistance across their programs for chronic disease prevention and health promotions [28].

### Stage 1: Time series clustering

The cluster solution is based on the daily number of newly reported, confirmed COVID-19 cases by county over time. No other information is used to determine the cluster membership. For time series clustering, there are three important decisions that affect the cluster solutions: (1) the scaling and preprocessing of the data; (2) the distance measure between clusters; and (3) the clustering algorithm. Liao [29] provides an accessible overview of time series clustering methods.

#### Scaling the data

For each county, the counts of newly reported confirmed COVID-19 cases were smoothed using a seven-day moving median to account for weekly seasonal patterns and reporting anomalies in the data. We rescaled the seven-day moving medians for each county so that the values fall between 0 and 1 as follows:

$$\text{MM}7_{t}^{\text{scaled}}=\text{max}(0,\;\frac{\text{MM}7_{t}}{\text{max}(\text{MM}7)}),$$

where **MM7** is the vector of all seven-day moving medians of newly reported confirmed COVID-19 cases for a given county and MM7_*t*_ is the observation from this vector at time *t*. Some counties reported negative cases on some days, resulting from reclassification of their recent cases: thus, it is possible that the moving median is negative in rare cases. This is why the max is required in the above equation. Rescaling the data in this way is done to focus the time series clustering on the pattern of the disease progression, not the magnitude of the daily case counts which may be dependent on many factors such as county size, population, etc.

#### Distance measure

In order to cluster the scaled time series profiles, it is necessary to measure the distance between the profiles. There are many ways to measure the distance between time series, including Euclidean distance, dynamic time warping [30], Pearson’s correlation coefficient and others [29, 31]. For this analysis, Euclidean distance was chosen specifically because it is computationally efficient and is not an elastic measure such as dynamic time warping. Euclidean distance provides a shape-based measure that gives one-to-one indexing across the profiles; thus, it preserves the time-based shape of the profiles which is desired in this application of time series clustering. The Euclidean distance between two time series, *r* and *s*, of length *T* is defined as

$$d_{E}\left(r,s\right)=\sqrt{\sum_{t=1}^{T}{\left(r_{t}-s_{t}\right)}^{2}}.$$

#### Clustering algorithm

A large number of clustering algorithms have been proposed in the literature, which have been studied and compared in the context of time series [29, 31]. For this analysis, *k*-means clustering was used. A possible limitation of *k*-means clustering approach for exploratory research is the number of clusters must be pre-determined. In addition, there have been many measures proposed to assess the validity of the cluster solution [32]. The R package NbClust [33], computes up to 30 cluster validity indices for cluster solutions of a variety of sizes. This allows the analyst to select the solution with the most homogeneity within the clusters, and provides a systematic method for selecting the optimal number of clusters in a data set. For this analysis the NbClust package was used with the *k*-means clustering method to select the optimal number of clusters.

The *k*-means solution meets the five recommended guidelines for reporting cluster analysis solutions given by Clatworthy et al. [34], which recommends reporting the computer program, similarity measure, clustering algorithm, decision criterion for number of clusters, and an evaluation of cluster validity. Our methods and results have provided the first four of these as well as evidence of cluster validity by developing an explanatory/predictive model exploring the relationship between exogenous variables and cluster membership.

### Stage 2: Modeling

The clustering method described above resulted in each county being assigned to one of three different clusters. A model that includes the county and state-level exogenous variables was fit both to describe the relationship between these variables and to predict cluster membership. Specifically, a multinomial regression model was fit because it balanced computational efficiency, predictive accuracy and ease of explanation.

The multinomial regression model estimates the relative probability of a county falling in each of the clusters given the value of the predictor variables. Let ***x***_*i*_ denote the vector of predictors for county *i* and ***β***_*k*_ denote the vector of unknown coefficients for determining the probability that county *i* falls in cluster *k*. Define

$$\frac{\pi_{ik}}{\pi_{ij}}=\text{exp}[\beta_{0}+x_{i}^{\prime }\beta_{k}]$$

where *β*_0_ is an intercept term. The *j*^*th*^ cluster is the arbitrarily chosen reference cluster (in our analysis, the reference cluster has been assigned to the cluster containing the largest number of counties). The model parameters were estimated in R using the multinom function from the nnet package version 7.3-15.

Prior to the application of the multinomial regression model, *k*-NN (*k*–Nearest Neighbors imputation, with *k* = 5) was used to impute missing observations for *Population Density* and *Government Response Index* (1 case), and *Percent Seniors* (36 cases) in our predictor variables. We used the *Gower* distance to compute the distances between the observation, with missing data, and the remaining counties (with complete data for the missing variables). The median value based on the 5-nearest neighbours was used to impute the missing values in *Population Density*, *Government Response Index*, and *Percent Seniors*. Our implementation utilized the VIM package in R [35]. We have elected to utilize this approach since it: (a) ensured that each of our predictor variables were compiled from a singular source, which ensures that the integrity of the data; and (b) allows us to explain/predict the cluster assignment for all 3,108 counties.

## Results

### Time series cluster solution

**Fig 5:**
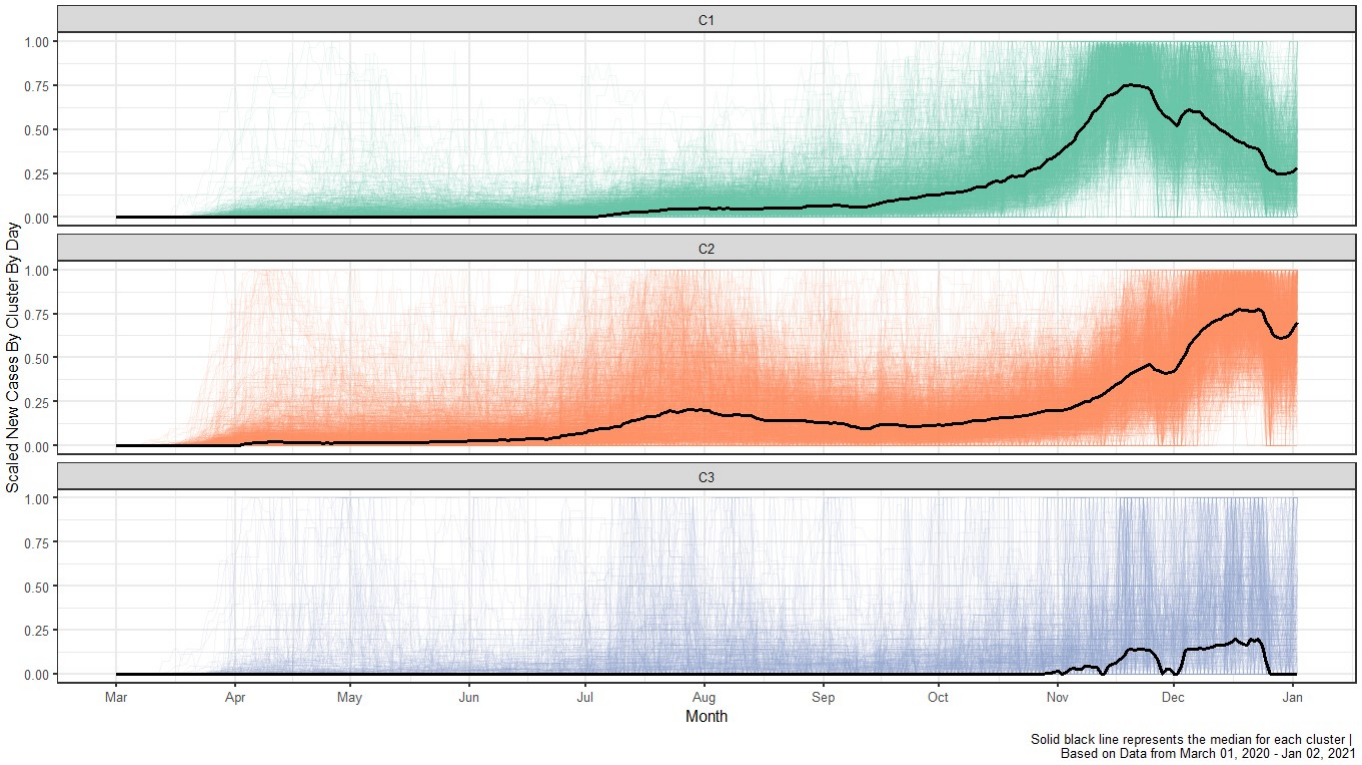
Map of the three scaled time-series profile clusters of COVID-19 cases by county in the contiguous United States.

**Fig 6:**
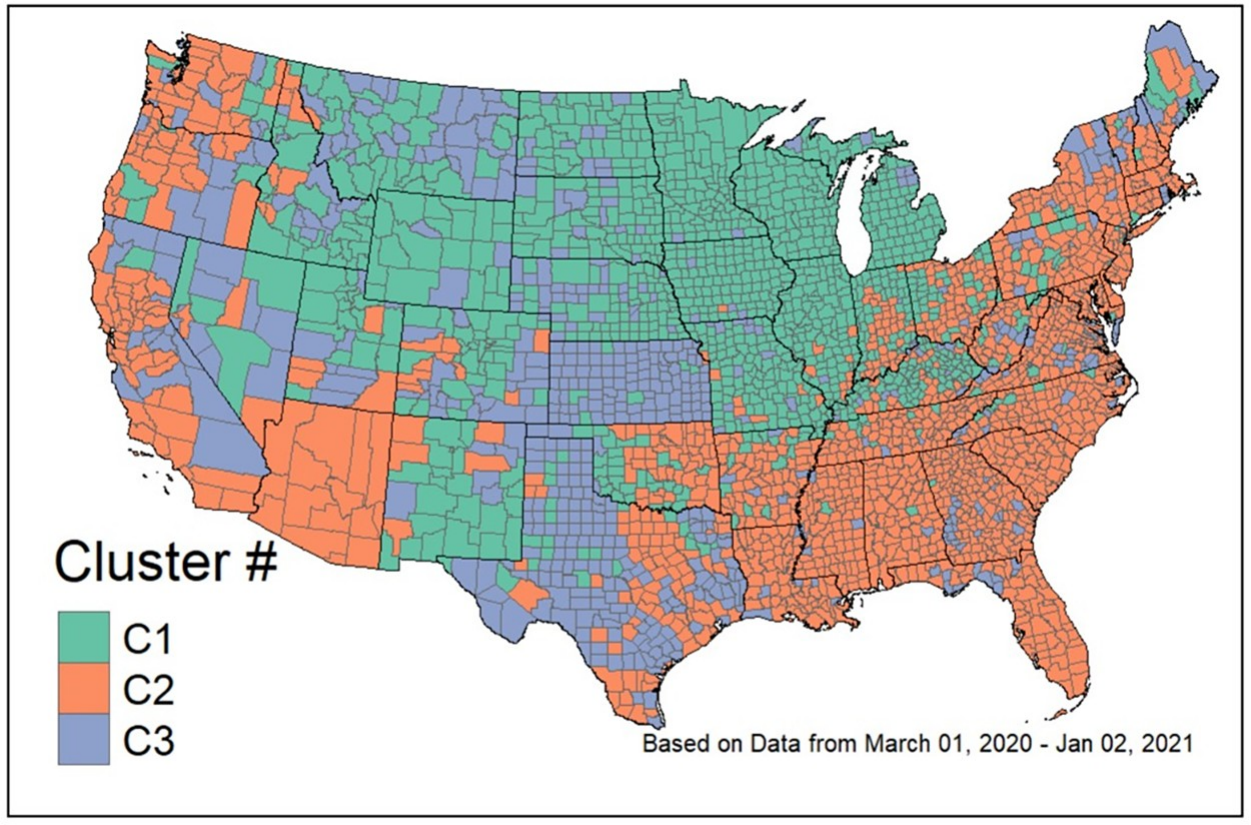
A spaghetti plot, where the median scaled time-series profile for each cluster is bolded and the remaining profiles within the cluster are shown in the background.

We computed *k*-means cluster solutions ranging from *k* = 2 to 49 clusters based on the scaled time series profiles of the daily confirmed COVID-19 cases. We evaluated twenty-six recommended cluster validity indices [32] for each *k*. For a full list of the cluster validity indices considered, see Charrad et al. [32]. Twelve of the twenty-six cluster validity indices indicate a three-cluster solution is preferred. The second most preferred solution is a four-cluster solution, which was selected by five out of the twenty-six indices. Based on majority rule of the validity indices, a three-cluster solution is retained. The geographic distribution of the clusters is shown in Fig 5. Furthermore, Fig 6 shows the time series profiles for each of the clusters.

The results of the cluster analysis provide an answer to our first research question: How many distinct clusters of counties exhibit similar COVID-19 patterns in the time-series of daily confirmed cases. Our results suggest that there are three distinct cluster patterns.

Fig 5 provides a map of the *k*-means three-cluster solution of the scaled time series profiles across the U.S. This map gives further insight into the geographic distribution of the clusters, which addresses our second research question: What is the geographic distribution of the counties within each cluster? The map shows that C1, which has 1,134 counties, is primarily distributed throughout the Midwest and Western United States. Fig 6 gives a visual summary of the cluster solutions. From this plot we see that most counties clustering in C1 experienced an outbreak that began gradually in the late summer, and increased substantially with many counties reaching a peak in the late fall of 2020. Counties in C1 include Hennepin County, MN which includes the city of Minneapolis and Nye County, NV, which is Nevada’s largest county in land area. The counties clustering in C2, the largest cluster with 1,380 counties, are located distinctly in the Eastern and Southeastern regions of the U.S. along with the West Coast and a few counties in the Southwest. From Fig 6 it is clear that many of these counties experienced an outbreak of COVID during the spring, a second wave that began in late summer, and a surge of cases in the fall that peaked shortly after the cut-off date for our analysis. Counties in C2 include, e.g., New York County, NY, which is the most densely populated county in the U.S. and consists mostly of Manhattan, and Navajo County, AZ, which contains over six thousand square miles of American Indian reservations. The smallest number of counties, 594, clustered in C3. These are located throughout the western states and in rural areas, sporadically throughout the U.S. Counties in C3 include Garfield County, MT, a county with one of the lowest population densities in the U.S. and Pike County, GA, a small county covering only 219 square miles in central Georgia. The outbreak activity in these counties seems to mostly flat, but increasing beginning in the late fall.

### Modeling results

Here, we provide the results of an explanatory model to answer our third research question: Are county-level demographic, socioeconomic and political variables associated with the COVID-19 case patterns?

Table 1 gives a summary of the predictor variables for each cluster. Note that these summaries exclude one missing observation for each of the *Population Density* and *Government Response Index* variables as well as 36 missing observations for the *Percent Seniors*. From this table, we see that C1 includes 41.9% of the *Rural/Underserved* counties and the majority of counties in *CDC Regions* E and H. Cluster C2, the counties experiencing an early outbreak in the spring of 2020, contain most of the counties not classified as *Rural/Underserved* and most of the counties in *CDC Regions* A, B, C, and D (the eastern coastal states in the U.S.), as well as many counties in I and J (the western coastal states). Cluster C3, the counties that only began to show an outbreak in late fall 2020 include a minority of the *Rural/Underserved* counties, and are spread throughout the U.S. Notably, C3 shows the lowest average *population density*.

**Table 1:** A summary of how the predictor variables are distributed per cluster. For each numeric variable, we report the mean, standard deviation (SD), first quartile (Q_1_), and third quartile (Q_3_). For categorical variables, we report the distribution of each subcategory across the three clusters. The row summation for a subcategory may deviate slightly from 100% due to rounding errors.

		C1 (*n* = 1, 134)				C2 (*n* = 1, 380)				C3 (*n* = 594)			
		Mean	SD	Q_1_	Q_3_	Mean	SD	Q_1_	Q_3_	Mean	SD	Q_1_	Q_3_
*Population Density* in log units		3.57	1.48	2.65	4.37	4.65	1.48	3.65	5.53	2.25	1.72	1.02	3.52
*Government Response Index*		45.67	8.19	38.89	50.67	49.57	7.05	45.28	53.61	46.84	7.60	40.83	49.17
*Percent Seniors*		24.98	5.11	22.00	28.00	23.78	4.99	20.78	26.20	26.92	6.63	22.2	31.18
*Poverty Percent*		13.44	5.43	9.90	15.60	16.27	6.34	11.7	20.1	15.96	6.06	12.00	18.30
		C1 (*n* = 1, 134)				C2 (*n* = 1, 380)				C3 (*n* = 594)			
		Percent				Percent				Percent			
*County Type*:	R/U	41.9%				29.1%				29.0%			
	Other	30.8%				60.5%				8.7%			
*CDC Region*:	A	10.1%				68.2%				21.7%			
	B	9.9%				77.3%				12.8%			
	C	2.2%				87.1%				10.8%			
	D	24.7%				69.8%				5.5%			
	E	78.0%				19.1%				2.9%			
	F	17.3%				47.9%				34.8%			
	H	62.9%				2.4%				34.7%			
	I	5.6%				66.7%				27.8%			
	J	32.8%				45.4%				21.8%			

The coefficients from the multinomial logistic regression along with the odds ratio (OR) are given in Table 2. The baseline category for analysis was chosen to be the cluster with the largest number of counties, which is Cluster 2 (C2). Each coefficient shows the linear change in the natural log of the odds ratio of a county classifying in the corresponding cluster as indicated by the column vs. the baseline cluster. Similarly, the OR shows the multiplicative change in the odds of a county classifying in the corresponding cluster as indicated by the column vs. the baseline cluster. For example, in the columns of Table 2, labeled C1, the first coefficient corresponds to the variable *County Type Rural/Underserved*. The coefficient, 0.644, suggests that, *ceteris paribus*, the odds of a county classifying in C1 vs. C2 is higher by a factor of OR = exp(0.644) = 1.904 when that county is *Rural/Underserved*.

**Table 2:** Results of multinomial logistic regression for the probability of falling in C1, and C3. Note that we have used C2 as the reference cluster since it contained the largest number of counties.

	Cluster Membership					
	C1			C3		
	$\hat{\beta }$	(95% CI)	OR	$\hat{\beta }$	(95% CI)	OR
*County Type: Rural/Underserved*	0.644	(0.358, 0.930)	1.904	0.341	(0.027, 0.654)	1.406
*Population Density*	−0.269	(−0.380, −0.159)	0.764	−0.969	(−1.102, −0.836)	0.379
*Government Response Index*	0.032	(0.013, 0.051)	1.033	0.061	(0.039, 0.082)	1.063
*Percent Seniors*	−0.018	(−0.043, 0.007)	0.982	−0.001	(−0.027, 0.024)	0.999
*Region B*	0.310	(−0.446, 1.066)	1.363	0.108	(−0.587, 0.802)	1.114
*Region C*	−1.263	(−2.235, −0.292)	0.283	−0.369	(−1.078, 0.340)	0.691
*Region D*	1.245	(0.518, 1.972)	3.473	−1.103	(−1.884, −0.322)	0.332
*Region E*	3.546	(2.854, 4.238)	34.674	−0.485	(−1.299, 0.330)	0.616
*Region F*	1.199	(0.439, 1.959)	3.317	0.501	(−0.207, 1.209)	1.65
*Region G*	5.401	(4.413, 6.390)	221.628	3.681	(2.699, 4.663)	39.687
*Region H*	4.436	(3.466, 5.406)	84.437	1.605	(0.618, 2.593)	4.978
*Region I*	−0.577	(−1.684, 0.531)	0.562	−0.800	(−1.625, 0.025)	0.449
*Region J*	1.649	(0.842, 2.455)	5.202	−0.484	(−1.336, 0.368)	0.616
*Poverty Percent*	−0.048	(−0.070, −0.027)	0.619	−0.019	(−0.042, 0.003)	0.981
Constant	−1.712	(−3.365, −0.059)	0.181	−0.407	(−2.176, 1.361)	0.666

Comparing C1 (late summer outbreak) to C2 (the baseline) which experienced earlier outbreaks, counties with higher *Population Density* and a higher *Poverty Percent* are associated with a lower odds of classifying in C1 (OR = .764 and.619). *Rural/Underserved* counties and those with a stronger *Government Response Index* are associated with a higher odds of classifying in C1 (OR = 1.904 and 1.033). In terms of geographic location, counties located in *CDC Regions D, E, F, G, H, J* were more likely to cluster in C1 compared to C2. These regions are primarily in the central United States, excluding the East Coast, California, Arizona, and Nevada. Counties in *Region C*, the Southeastern Atlantic states, were less likely to cluster in C1.

Comparing C3, which has experienced limited cases as of this writing, to C2, increased *Population Density* and location in *CDC Region* *D*, the deep South are associated with decreased odds of classifying in C3 (OR = .379 and OR = .332). *Rural/Underserved* counties and a higher *Government Response Index* along with location in *CDC Regions* *G* and *H* (comprised of western states), are associated with a higher odds of classifying in C3.

Table 3 shows the counts and percent accuracy of predictions for each of the clusters based on the explanatory/predictive model. For each cluster, the largest predicted category is the true cluster; however, clusters C1 and C2 were easier to identify than C3.

**Table 3:** A summary of the predictive performance of the multinomial regression model. For a given cluster, the first and second rows show the number and percentage of predicted cases, respectively.

*Cluster*	*Predicted Cluster*			*Total*
	C1	C2	C3	
C1	835	130	151	1116
	74.8%	11.6%	13.5%	
C2	224	1191	202	1617
	13.9%	73.7%	12.5%	
C3	75	59	241	375
	20.0%	15.7%	64.3%	

**Fig 7:**
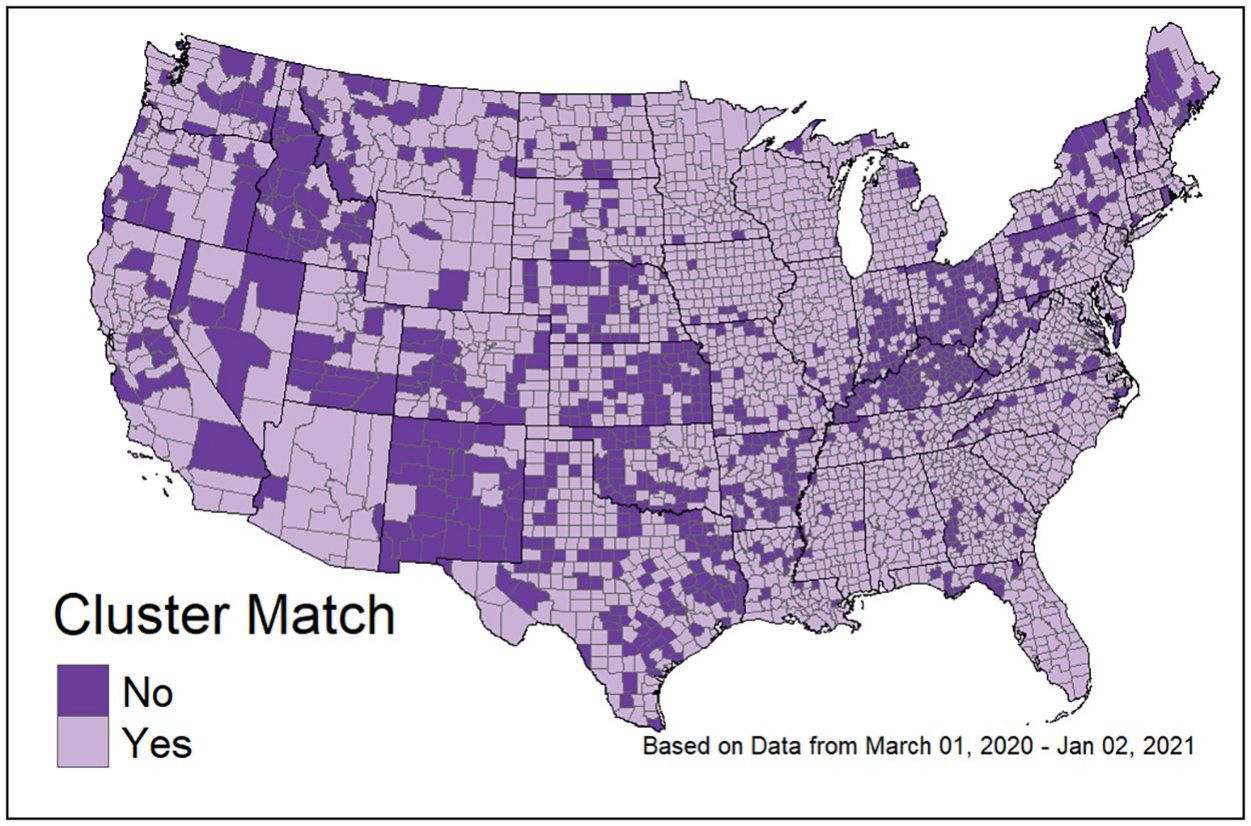
Map of the prediction accuracy of the multinomial logistic model describing the time series cluster solution. Counties in light purple (labeled Yes) were correctly classified with the model. Counties in dark purple (labeled No) were incorrectly classified.

Fig 7 shows the geographic distribution of the accuracy of the multinomial logistic model in predicting cluster membership. Counties that are accurately predicted are shown in light purple, while counties that are not accurately predicted by the model are shown in dark purple. From this map it is clear that additional data are needed to describe the outbreak patterns throughout the U.S.

## Discussion

Although there have been several country-level and even regional level analyses of the outbreaks, the county-level analyses of COVID-19 in the U.S. have focused on the relationship between income and demographic variables and the case counts (see, e.g., [3, 9, 10]). Our results show that there are distinct patterns, and many counties exhibit a similar “signature” in the outbreak pattern. As shown in Fig 5, there is a substantial geographic component to these outbreak patterns. The results of the explanatory model further illustrate the relationship between the region of the country and the outbreak patterns.

The large effects associated with certain regions illustrate the importance of the regions in classifying counties into the clusters. However, the geography alone does not explain the signature patterns in the disease outbreak. For example, consider counties that clustered C3, which showed a relatively flat pattern of cases that slowly began rising in late fall 2020. Although mostly centered in the western U.S., we see these counties scattered throughout the North and Southeastern U.S. Further, we see scattered incidences of counties with late summer outbreaks as well as early spring outbreak patterns throughout the U.S. This, coupled with the explanatory model suggest that there is information in addition to geography that is useful in understanding a regional COVID-19 outbreak pattern.

Our multinomial logistic regression model is developed to explain, not COVID-19 cases, but the pattern of outbreaks across U.S. counties. This is a unique approach to understanding how certain demographic, socioeconomic, and political variables relate to the pattern of outbreaks across the U.S. While our results confirm a large geographic effect, our model also shows other important variables relate to the outbreak patterns.

We are not the first to consider the relationship between the rural nature of a region and the COVID-19 pandemic. While some research and commentary suggest that negative outcomes are higher in these underserved areas (see, e.g., [4], and [5]), our results show that counties deemed rural or underserved by the Consumer Financial Bureau experienced a later outbreak pattern, classifying in the late summer outbreak (C1) or the late fall outbreak (C3) with higher probability than in the early spring outbreak pattern (C2). These results support the July, 2020 report of Bishop [3] who suggested that a higher proportion of urban counties compared to rural ones were experiencing high case counts and high test positivity rates for COVID-19.

The negative association between *Population Density* and the later outbreak signatures of C1 or C3 vs. the early outbreak pattern of C2 is also an interesting finding. Several studies have confirmed the relationship between population and disease spread [36–38]. Some have also considered the effect of connectedness of the population through airports, transportation, and other transmission opportunities on disease spread [38–40]. Because larger metropolitan areas tend to be both more connected and allow more opportunity for interaction, it is difficult to disentangle the opportunity for contact with the density of the population in understanding disease outbreak patterns.

Although it is well known that the COVID-19 outcomes such as hospitalizations and deaths are positively associated with age [7], the relationship between age of the population and disease spread is less understood. While it is believed that seniors are more susceptible to contracting COVID-19 once exposed [8], counties with higher proportions of seniors have been shown to have lower incidences of outbreaks [9]. Our study considers the relationship between age and the pattern of outbreaks and did not confirm a significant relationship between the age of the population and the outbreak patterns.

Similarly, researchers have considered the relationship between poverty and both outcomes and cases of COVID-19. Evidence of the relationship between poverty and case counts has been weak or explained by other factors such as state-level effects [9]. In our study, counties with a higher *Poverty Percent* are also slightly less likely to experience a late summer pattern of outbreaks compared to an early spring pattern. Poverty Percent was not a significant predictor of membership in the early pattern of outbreaks compared to counties with a mostly flat pattern of outbreaks. Although our model is not causal in nature, the overall profile of counties with early vs. late outbreak patterns suggests that those in the early outbreak pattern were more densely populated, less rural, and in regions of the country that are associated with larger metropolitan areas.

The government response to the COVID-19 pandemic has been hotly debated in the media, and many feel that the government response is important in containing the spread of the disease. We included the the *Government Response Index* developed by the Blavatnik School of Government [14] in our model to assess the relationship between this important variable and the outbreak patterns. The index consistently accounts for school closures, testing, contact tracing, and economic response. Although our study considers the patterns of outbreaks, not growth rates or disease counts, our results show a small but significant relationship between classifying in C1 or C3 vs. C2. This suggests that, after controlling for regional and demographic factors, stronger state government containment measures are related to a later outbreak pattern at the county level. This finding is consistent with research findings in Ficetola and Rublini [2] and Islam et al. [15] which show that stronger containment measures reduced growth rates of the disease outbreak.

## Limitations

The observational analysis presented in this paper is time dependent, and was conducted as the pandemic continues to emerge globally. The retrospective, observational nature of this study makes it impossible to infer causation from the relationships shown in our model. Further, the outbreak patterns are certain to change over time as new containment policies are adopted nationwide and vaccines for COVID-19 continue to seek approval and distribution globally.

The purpose of our multinomial regression model was to explain the associations between socioeconomic, political and demographic variables, and the clustering of time-series profiles of the 3,108 counties. Hence, we do **not** imply that the relationship between government restrictions and outbreak patterns is causal. Since our model seeks to explain the cluster membership, not case counts, we cannot infer what factors lead to higher (or lower) rates of COVID.

The Government Response Index is a state-level predictor that it is constant across all counties in the state. Using a predictor at the state level in a model to explain cluster membership at the county level could lead to an ecological fallacy in the Conclusions. However, because local governments such as counties in the U.S. must be granted authority from the states [41], the state-level variable provides important information in determining the response of localities within the states. Ideally the model would contain both state-level and county-level predictors related to the government response. Unfortunately, county-level information regarding the government response is not consistently available for most counties in the U.S. The absence of county-level information on the government response is a limitation of the study.

Finally, there may have been some exceptions to our statement that the Government Response leveled off to a constant state during the study period. For example in Florida, there were substantial temporal changes in the value of the index which might have contributed to outbreak patterns.

## Conclusions

As of the end of 2020, our results retrospectively illustrate the emergence of clusters of outbreak patterns. Although there are strong geographic determinants of the patterns, there are also several demographic, socioeconomic and political variables that are shown to relate to the pattern of outbreaks across the U.S. counties. It is important to note that there are other important socioeconomic and demographic variables that may explain the cluster of disease outbreak in the U.S., but this research shows that (a) patterns of outbreaks can be grouped together into three main clusters; and (b) membership in these pattern groups is related to variables in addition to geography.

Interestingly, many of the variables that are significantly related to the outbreak patterns are, in the short run at least, outside the control of a state and local government. Neither the region, the population density, nor the poverty level of a county are within the span of control of the government, but all of these variables were related to the outbreak patterns. Stronger government response, however, is within control of state governments and was associated with patterns of outbreaks occurring in late summer or late fall relative to early spring of 2020.

The local patterns in outbreaks suggest that decisions regarding the timing of mitigation efforts should be informed by local conditions. Local conditions vary across the country and even within each state, and the clusters of patterns exhibit spatial distributions. By understanding the patterns of COVID-19 progression across the country, policy and mitigation standards can benefit from regional information at a given time in order to better preserve public health.

All the data capturing, processing, visualization, and analysis were performed in R [42] version 4.0.3. To facilitate the reproduction of our research, we have capitalized on the R Markdown documentation mechanism to produce an automated report that contains all our data, analysis and results, which we host at https://fmegahed.github.io/covid_analysis_final.html, following the best practices of Jalali et al. [43] in reporting and documenting analyses for COVID-19.
